# Editorial: Plant Microbiome: Interactions, Mechanisms of Action, and Applications

**DOI:** 10.3389/fmicb.2021.706049

**Published:** 2021-07-05

**Authors:** Alok Kumar Srivastava, Prem Lal Kashyap, Gustavo Santoyo, George Newcombe

**Affiliations:** ^1^National Bureau of Agriculturally Important Microorganisms (ICAR), Mau, India; ^2^Indian Institute of Wheat and Barley Research (ICAR), Karnal, India; ^3^Instituto de Investigaciones Químico-Biológicas, Universidad Michoacana de San Nicolás de Hidalgo, Morelia, Mexico; ^4^Department of Forest, Rangeland and Fire Sciences, University of Idaho, Moscow, ID, United States

**Keywords:** plant growth-promoting rhizobacteria, sustainable agricultural applications, plant-microbe associations, bacterial communities, plant-fungal interactions, plant microbiome, arbuscular mycorrhiza

An improved understanding of the plant microbiome is likely to yield valuable applications in agriculture, horticulture, forestry, and in conservation of natural plant communities. The symbiotic microbiota of plants are involved in everything from nutrient acquisition to escalation of defense systems during periods of biotic and abiotic stresses. A large body of research has shown that the interactions between plants and their microbiomes are highly complex and dynamic in nature (Abhilash et al., 2016; Compant et al., 2019; Srivastava et al., 2021). Biotic and abiotic stresses represent a continuous and prime threat to global food and fiber production (Dean et al., 2012; Santoyo et al., 2017; Begum et al., 2019; Gamalero et al., 2020). Our goal is to maintain plant productivity by managing the microbiome so that biotic and abiotic stresses are minimized. This research focus is yielding insights that should lead to better crop plant management, especially in resource-limited agricultural systems (Begum et al., 2019; Gamalero et al., 2020; Vescio et al., 2021). In other words, the successful implementation of microbiota-mediated crop protection will depend on the mechanistic understanding of how microorganisms interact with their hosts and with one another in natural environments. Considering these developments, our Research Topic “Plant Microbiome: Interactions, Mechanisms of Action, and Applications,” offers a timely snapshot of 25 articles on plant microbiome research with a special focus on specificity, diversity and function of complex microbial communities associated directly or indirectly with the plant (i.e., endophytic, epiphytic, and rhizospheric communities). Included is the translation of molecular understanding of plant microbiome research. Overall, we propose novel strategies and applications of growth-promoting microbes, arbuscular mycorrhizal (AM) fungi, and bioactive compounds in order to enhance resilience in the face of biotic and abiotic stressors.

The arbuscular mycorrhizal (AM) symbiosis is an ancient mutualism with global significance for more than 80% of all plant species and for their dependent communities. In this context, and in this special issue, Zhang et al. reveal that *Rhizophagus irregularis* inoculation improves plant growth by enhancing AM symbiosis, stimulating antioxidant response, and inhibiting lead uptake. They report melatonin as a potent regulator of plant growth that strengthens AM symbiosis and heavy metal tolerance. Ding et al. expand our knowledge of the regulation of plant–pathogen relationships by examining the antagonistic effect of the AM fungus (*Sieverdingia tortuosa*) against the anthracnose pathogen (*Colletotrichum lentis*) of common vetch (*Vicia sativa* L.). The AM fungus activated phenylpropanoid biosynthesis and the mitogen-activated protein kinase (MAPK) signaling cascade to strengthen the plant defense system against pathogen invasion. Besides this, the work of Bukovská et al. illustrate the role of AM fungi as efficient recyclers of nutrients bound in organic forms from soil to plants.

Diazotrophic bacteria are important components of many microbiomes. They have the potential to penetrate the internal tissues of plants and work as endophytic, mini-nitrogen factories (Imran et al.). Additionally, they also contribute to plant growth promotion and biological control as revealed by the study of Singh, Singh, Guo et al.. The latter analyzed and identified the genes involved in growth promotion and biocontrol of fungal pathogens in the sugarcane, root-associated endophyte (*Pseudomonas aeruginosa* strain “B18”). Similar insights were provided by Singh, Singh, Li et al. who documented the role of diazotrophic bacteria (i.e., *Pantoea dispersa* and *Enterobacter asburiae*) in growth promotion of sugarcane by inducing nitrogen uptake and defense genes. Likewise, Solanki et al. shed light on microbial composition, distribution, and dynamics of diazotrophs in the sugarcane legume-intercropping system; sugarcane intercropping with legumes and short-duration vegetable crops boosted soil fertility and biological nitrogen fixation without any negative impact on crop production. Similarly, Choudhary et al. explored topsoil bacterial community and nutrient dynamics under cereal-based, climate-smart, agri-food systems. It was clear that microbes wield a positive influence on soil resilience in terms of nutrient cycling and availability to plants.

In forest ecosystems, microbial communities, particularly fungal communities, have been documented with a metabarcoding approach. Environmental factors (e.g., seasonal and soil properties) and host tree species have been explored as possible determinants of community structure (Park, Oh, Yoo, Fong et al.). Another study by Park, Oh, Yoo, Park et al. portrayed the composition and the interaction of the root fungal microbiome of *Pinus densiflora* with [the valuable] pine mushroom (*Tricholoma matsutake*). The authors concluded that successional change plays an important role in fungal microbiome composition in pine seedlings during transplantation and seedling growth stages. *Tricholoma matsutake* is an ectomycorrhizal fungus that produces edible mushrooms. But not only abiotic factors or properties modulate the plant microbiome; soilborne fungal pathogens can as well. For example, *Phytophthora cinnamomi* not only causes root rot disease in avocado trees but it also can modify the composition of the rhizosphere microbiome by increasing the abundance of opportunistic fungal pathogens (Solís-García et al.). Similarly, Chen et al. noted a process in which the diversity of the foliar microbiome was reduced. Interestingly, Newcombe et al. revealed that plants may host pathogens of their competitors. One pathogen in particular, *Fusarium culmorum*, was present as an endophyte in asymptomatic leaves of wild cottonwood trees while retaining the capacity to cause disease in wheat of the region. There has been little investigation into microbiome effects on plant competition, perhaps in part because competing plants in agricultural fields are controlled mechanically or chemically.

Barajas et al. explored the role of soil in microbiome structuring by employing a two-step model of plant root microbiome acquisition under multiple plant species and soil sources. They concluded that plant domestication trade-offs drive tomato and ruderal metagenomic differences and that they may even be the deciding factor for plant-bacteria interaction outcomes. Using the same model plant (i.e., tomato), Haque et al. highlight the advantages of biofilm-producing bacteria in alleviating water stress along with plant growth promoting attributes and disease suppression capabilities. Silva et al. focused on phenotypic and genomic aspects of the potential phosphorus-solubilizing microorganisms as bioinoculants in agriculture. Sorokan et al. examined the role of endophytic *Bacillus* spp. as biocontrol agents for the management of viruses and their arthropod vectors. They suggest that the endophytic lifestyle, RNase, and antifeedant activity of *Bacillus* spp. should be the baseline for the development of bio-inoculants for protecting crops from viral infection. Similarly, in the case of phytoparasitic nematodes, Banakar et al. focused on the FMR Famide-like peptide (FLPs) family of neuromotor genes and demonstrated the potential management of *Meloidogyne incognita* causing root knot diseases in plants.

The complete sequencing and genome analysis of plant growth promoting bacteria are revealing extensive mechanisms for stimulating plant growth and protecting against pathogens, as supported by the work of Singh, Singh, Li et al. The authors determined the genome of *Enterobacter roggenkampii* ED5, a nitrogen-fixing endophytic bacterium with biocontrol and stress tolerance properties. Other work by Li et al. also revealed new genomic characteristics of the symbiotic strain *Bradyrhizobium diazoefficiens* 113-2. Additionally, its genome was compared with other related genomes providing new molecular insights into species specificity and host specificity. Another work by Singh, Singh, Li et al. deciphered the complete genome of the endophytic bacterium *Pseudomonas aeruginosa* strain “B18,” whose beneficial interactions with plants include direct and indirect mechanisms of plant promotion.

Beside research articles, new perspectives and reviews enrich this special issue. For instance, Oyserman et al. propose that genotype, environment and microbiome (GEM) interactions shape host phenotypes. They outline the versatility of the GEM model, and thus provide a way to keep in view the important role of the microbiome in determining plant fitness. Similarly, Imran et al. reviewed the role of diazotrophs and their important activity in nitrogen fixation, and how these free-living organisms can help to reduce nitrogen inputs to agricultural systems. Likewise, Kaul et al. highlighted the concept of engineering plant microbiomes for enhancement of plant functions such as combating physiological stresses, as growth promoters, acquisition of essential nutrients, priming host plant defenses, increasing niche breadth, biocontrol agents and in biogeochemical processes. Other reviews of Ray et al. and Babalola et al. provide an overview of how plant performance is influenced by microbiome diversity and function; various holistic microbiome approaches for enhancing crop productivity and restoring soil health are discussed. Naamala and Smith provide an elaborate review of the identification, characterization and application of compounds of microbial origin. They suggest ways to move forward with inoculants to improve agricultural practices.

There is no doubt that in the coming decades the twin challenges of feeding a growing human population while conserving biological diversity will grow. To be successful in meeting these challenges, the plant microbiome will need to be better understood and managed. The articles of this special issue show us where we stand with the plant-microbe interactome. Novel approaches and innovative perspectives point to the exciting possibilities of new insights and applications. Well-executed, agricultural field trials may still be needed. But, there is enough knowledge already to inspire researchers to increase efforts to decode the role of the microbiome in plant performance and health.

## Author Contributions

All authors listed have made a substantial, direct and intellectual contribution to the work, and approved it for publication.

## Conflict of Interest

The authors declare that the research was conducted in the absence of any commercial or financial relationships that could be construed as a potential conflict of interest.
